# Autonomous optimisation of biocatalytic reactions: enzymatic synthesis of *N*-benzyl acetoacetamide in continuous flow

**DOI:** 10.1039/d5sc04249f

**Published:** 2025-09-10

**Authors:** Matthew J. Takle, Sebastian C. Cosgrove, Adam D. Clayton

**Affiliations:** a Institute of Process Research and Development, School of Chemistry, School of Chemical Process Engineering, University of Leeds Leeds LS2 9JT UK A.D.Clayton@leeds.ac.uk; b School of Chemical and Physical Sciences, Centre for Glycoscience, Keele University Keele ST5 5BG UK

## Abstract

Biocatalysis represents an invaluable tool for developing more sustainable pharmaceutical manufacturing processes. However, the optimisation of these complex transformations remains challenging when using traditional methods. Recent advances in the field of chemical reaction optimisation have employed Bayesian optimisation, which has been successfully integrated into automated flow reactors to develop self-optimising reactor platforms. Although self-optimisation has been applied to chemical transformations with great effect, it has not yet been applied to biocatalytic reactions. In this work, we report the first application of multiobjective and mixed variable Bayesian optimisation for the automated development of a biocatalytic transformation. Using this approach, we develop a significantly improved process for the direct amidation of a β-ketoester in just 31 hours of experimental time, while also extracting insights from the black-box models to understand solvent-dependent effects and interactions among the reaction conditions.

## Abstract 2

The first reported application of Bayesian optimisation for autonomous optimisation of a biocatalytic reaction in continuous flow. Significantly improved the selectivity, productivity and sustainability for the synthesis of a β-ketoamide.

## Graphical abstract

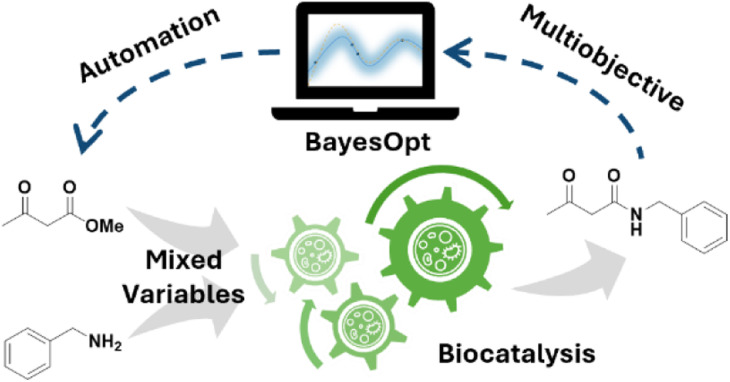

## Introduction

Biocatalytic transformations deliver high levels of enantio-, chemo- and regioselectivity under mild reaction conditions compared to chemical transformations.^1^ Their ability to create more direct synthetic routes while consuming less energy and reducing environmental impact has led to their increased application in the pharmaceutical industry.^2–4^ Biocatalysis is now routinely implemented in continuous flow owing to the numerous benefits that it affords, such as; increased reaction rates from higher effective loading within packed bed reactors (PBRs), and longer life spans of enzymes due to the reduced mechanical stress applied in flow.^5–8^ This forms a synergistic combination to develop sustainable and scalable industrially relevant processes. However, the optimisation of biocatalytic reactions can be extremely challenging owing to the large number of mixed variables, *i.e.* continuous (concentration, co-factor stoichiometry, buffer concentration, pH, temperature *etc.*) and categorical (buffer type, co-solvent *etc.*), associated with them. This creates a huge number of combinations of reaction conditions, with many complex interactions at play, that are extremely challenging to elucidate using classical optimisation strategies. Automation and data-led optimisation strategies are therefore well placed to tackle these challenges.^9–11^

Automation of biocatalytic reactions has been successfully employed by Niemeyer *et al.*, who demonstrated use of in-line NMR to monitor the reduction of 5-nitrononane-2,8-dione using alcohol dehydrogenase (LbADH) in real-time.^12^ The automated system detected decreases in conversion, as the enzyme deactivated over time, and adjusted the flow rate to maintain consistent performance. However, this approach was limited to adjusting only a single variable, when generally complex reaction systems are multivariate and rely on the interactions between variables to maximise performance. Furthermore, despite illustrating pioneering use of PAT tools and automation in flow biocatalysis, this system was only applied to process control, rather than optimisation.^13^

Currently, to the best of our knowledge, research into the automated optimisation of biocatalytic transformations has been limited to a single publication by Gruber-Woelfler *et al.*^14^ This notable work demonstrates effective use of automated design of experiments (DoE) to optimise the space-time yield (STY) of coumaric acid decarboxylation *via* a phenolic acid decarboxylase (bsPAD) enzyme in deep eutectic solvents.^15^ During this optimisation, three continuous variables (dilution ratio, residence time and temperature) were explored, using an iterative fractional factorial central composite design (CCD) approach. Although a categorical variable, the type of ceramic inset, was investigated, it was not handled within the automated DoE. Rather, the results of four different DoEs, one per inset, were evaluated retrospectively to ascertain which was best performing.

DoE significantly reduces the number of experiments required compared to traditional one-factor-at-a-time methods, while enabling greater insight into interactions between reaction parameters. However, DoE is a non-adaptive technique, typically implemented through predefined experimental campaigns. As a result, if the initial understanding of the reaction space is poor, many data points may fall at the boundaries of the output space (*e.g.*, 0% or 100% conversion), offering limited information and requiring manual redesigns. Additionally, DoE suffers from scalability challenges. For example, for commonly used two-level factorial designs, the number of required experiments grows exponentially with the number of variables, *n* (*i.e.*, 2^*n*^), making it impractical for high-dimensional problems.

Bayesian optimisation (BO) enables efficient exploration of a reaction space by iteratively updating a surrogate model, usually Gaussian processes (GPs), to predict outcomes and guide subsequent experiments. In contrast to DoE, which samples the parameter space more uniformly, BO uses an acquisition function to strategically focus on the most promising regions, while still exploring uncertain areas. This adaptive approach refines the model with each new data point, often identifying global optima with fewer experiments, thus addressing scalability limitations. However, similar to DoE, BO does still have scalability issues, with successful applications typically limited to problems with fewer than 10–20 parameters.^16^ Nevertheless, BO can more naturally accommodate mixed variable types and model non-linear response surfaces more effectively, as it uses kernel-based methods rather than relying on low-order polynomials.^17^

Combining BO with automated flow reactors has become increasingly implemented in process chemistry, in both academia and industry.^18^ This combination creates a highly efficient workflow that simultaneously decreases the number of experiments, experimental time, and the amount of material consumed to reach the global optimum of a chemical process.^19^ All of these are extremely important considerations when working in a time sensitive research field, such as the pharmaceutical industry, where the substrates can be extremely expensive and limited during early development. However, this methodology has not yet been applied to biocatalytic reactions.

Herein we outline the first autonomous optimisation of a biocatalytic reaction using BO algorithms for the synthesis of *N*-benzyl acetoacetamide in continuous flow. This work describes a multiobjective optimisation workflow, exploring both continuous and categorical variables, to understand the trade-off between yield and selectivity.

## Results and discussion

**Scheme 1:**
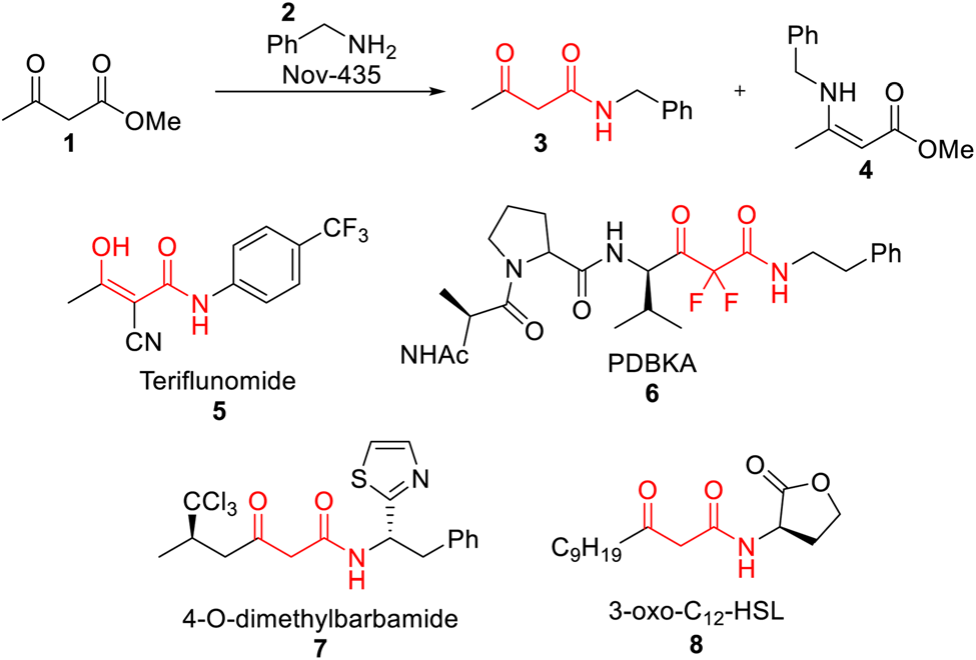
Nov-435 mediated synthesis of *N*-benzyl acetoacetamide 3 and examples of β-ketoamides in bioactive molecules.

This work focused on the direct synthesis of β-ketoamides from β-ketoesters *via* a Novozym-435 (Nov-435) mediated amidation reaction (Scheme 1). The synthesis of β-ketoamides is of great interest to the pharmaceutical industry as they are ubiquitous in bioactive molecules (Scheme 1) due to their multiple points of interaction within one functional motif.^20^ Although a relatively simple transformation, the greater reactivity of ketones compared to esters poses a significant chemoselectivity challenge, resulting in undesired enamine formation. To negate this, protecting groups can be used to attenuate the reactivity of the ketone, before the amide bond is formed. However, this adds multiple steps and generates excessive waste.^21^ The use of Nov-435, widely used for the kinetic resolution of alcohols and amines, can be used to drive the selectivity of this transformation through activation of the ester, allowing the amidation reaction to occur preferentially.^22–25^

The direct synthesis of *N*-benzyl acetoacetamide 3*via* CALB, a non-proprietary version of Nov-435, has previously been reported by Gotor *et al.* in 1993 delivering an 89% yield (Table 1, entry 1).^24^ However, more recently Lavandera *et al.* reported the same reaction to deliver a <20% yield (Table 1, entry 2).^25^ Although direct synthesis of 3 has been achieved using classical chemical transformations (Table 1, entries 3–6), these routes are either low-moderate yielding, use hazardous reagents or require harsh reaction conditions, making them unattractive routes to pursue, particularly on an industrial scale.^26–29^

**Table 1:** Examples of the direct synthesis of *N*-benzyl acetoacetamide, 3. STY = space-time yield, a measure of reactor productivity, defined as the amount of product formed per unit reactor volume per unit time (see SI eqn (S5))

Entry	Reagents	Conditions	Yield (%)	STY (g L^−1^ h^−1^)	Ref.
1	CALB	Dioxane, 30 °C, 18 h	89	1.18	Gotor, 1993 (ref. 24)
2	CALB	Dioxane, 60 °C, 48 h	<20	0.080	Lavandera, 2019 (ref. 25)
3	Neat	PhMe, 80 °C, 10 h	8	0.244	Roucoux, 2010 (ref. 26)
4	[BMIm]OH (100 mol%)	PhMe, 120 °C, 3.5 h	68	105	Maiti, 2016 (ref. 27)
5	HFIP (10 equiv.)	Neat, 80 °C, 12 h	79	12.0	Malakar, 2020 (ref. 28)
6	PEG 300	Neat, 120 °C, 1.5 h	75	71.6	Gaddamanugu, 2014 (ref. 29)
7	Nov-435	2-MeTHF, 30 °C, 8.4 min	94	274	This work

The previous works by Gotor and Lavandera were exclusively explored in batch. We expected the transition of this reaction into flow would deliver greater productivity (STY) and selectivity, owing to an increased rate of the catalysed amidation, through higher effective loading within the PBR, without increasing the rate of the competitive uncatalysed condensation reaction. Furthermore, the superior ability of flow reactors to control residence times, heating and mixing was anticipated to enhance selectivity and reproducibility. However, transitioning from batch to flow has associated challenges, including the requirement for all reaction components to be soluble at productive concentrations, which often involves significant redevelopment from reported batch conditions. Herein we aimed to rapidly optimise a highly selective, direct biocatalytic synthesis of *N*-benzyl acetoacetamide in flow using automation and BO algorithms.

### Self-optimising flow reactor

**Fig. 1:**
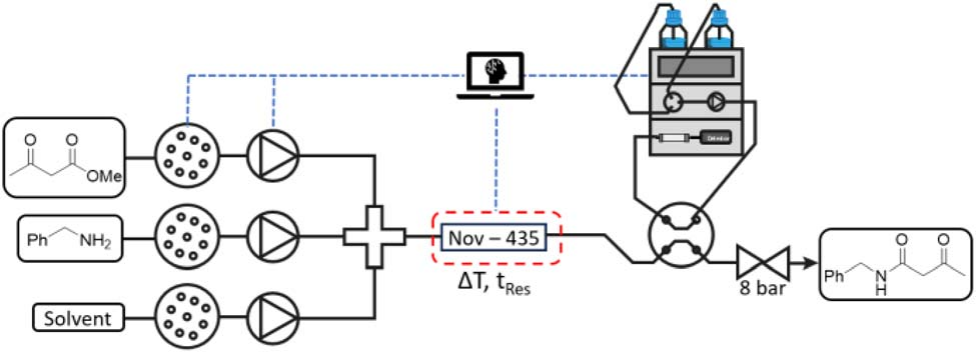
Self-optimising flow reactor controlled *via* MATLAB code.

During the optimisations the concentration of 1, stoichiometry of 2, residence time, temperature and solvent were investigated. A flow reactor system was assembled (Fig. 1), using two HPLC pumps to deliver the reagents and a third as a dilution pump, required to vary and control the concentration. Each pump was connected to a 16-port multiposition valve that delivers the reagents in the desired solvent. The three streams, connected by a crosspiece, delivered the reaction mixture to a PBR containing Nov-435. The PBR was housed within a stainless-steel heating jacket, heated by a PID controller. The product stream was passed through a sampling valve, where an aliquot of reaction mixture was directed to an on-line uHPLC. The whole system was pressurised by an 8 bar back pressure regulator, to maintain constant pressure. The HPLC pumps, PID controller, sampling valves and uHPLC spectra analysis were all automated through MATLAB code (see SI for equipment details and optimisation workflow).

Two algorithms were used for separate optimisations in this work. For multi-objective optimisation of continuous variables, Thompson Sampling Efficient Multi-objective Optimisation (TSEMO) was used, which employs GP surrogate models to determine an approximation of the true Pareto front.^30,31^ For single objective optimisation of mixed variables (*i.e.*, including solvent as a categorical variable), Adaptive Latent Variable Bayesian Optimisation (ALaBO) was used, which employs 2D latent variable GP modelling to create continuous representations of categorical variables.^17,32^ In both cases, the target of the optimisations was to maximise product 3 formation and minimise impurity 4 formation, achieved by using normalised peak areas as inputs to the algorithms. Herein, figures are plotted using these normalised values, and actual conversions provided in the SI.

### Novozym-435 stability study

The bounds of the reaction conditions under investigation were 200–350 mM of 1, 1–4 equivalents of 2, 1–10 minutes residence time, and 25–60 °C. Additionally, different solvents (MeCN, 2-MeTHF, anisole and dioxane) were explored as categorical variables. The concentration was derived from the original work by Gotor *et al.* where 125 mM was employed, therefore a higher target concentration range was selected to improve the STY of the process.^24^ The equivalents of 2 and the residence time were set within reasonable ranges to ascertain their relationships with the formation of product 3 and impurity 4. Finally, the upper limit of the temperature of 60 °C was set to fall below the thermal limit of the enzyme.

Investigating alternative solvents to dioxane, deemed to have “major issues” by the GSK green solvent selection guide while also possessing significant health risks, was of high priority.^33^ Many of the recommended green solvents contain functionalities that would create competitive reaction pathways (alcohols, esters or ketones). Therefore, MeCN, 2-MeTHF and anisole were selected for their improved green chemistry metrics, high availability, relatively low cost and lack of reactive functionalities.

The optimisation requires the enzyme to remain stable throughout the duration of the operating time to allow the algorithm to accurately evaluate the relationship between the inputs and outputs. To ensure this, a stability study was performed using the most forcing reaction conditions that could be generated during the optimisation, which typically results in the greatest amount of catalyst deactivation. This corresponded to the upper limits of each of the continuous variables (350 mM, 4 equiv., 10 min and 60 °C). These conditions were run for three hours in each solvent (MeCN, 2-MeTHF, anisole and dioxane), for a total duration of 12 hours, before repeating the cycle. This would evaluate the stability of the enzyme in each of the four solvents over a 24 hour period. Cycling between the solvents provided repeats to check for deactivation. The concentration of product 3, the catalytic product, was monitored to evaluate the enzyme activity.

**Fig. 2:**
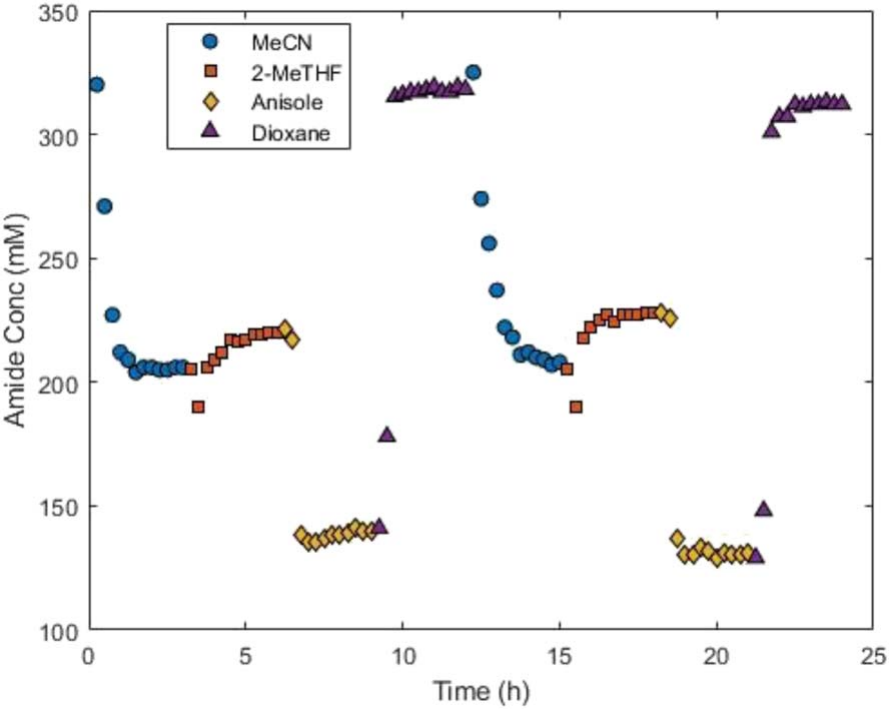
Solvent stability analysis at 350 mM, 4 equiv., 10 min and 60 °C, with the solvent varying between MeCN, 2-MeTHF, anisole and dioxane every 3 hours for 24 hours.

The stability of Nov-435 was successfully maintained over the course of the 24 hour experiment (Fig. 2). For all four solvents, the concentration of product 3 was consistent between the first and second round of experiments. This gave a high degree of confidence that no significant catalyst deactivation would occur throughout the duration of the optimisation. This experiment also demonstrated the significant effect that solvent was expected to play in this transformation, shown by the differing levels of amide production across all four solvents under the same set of reaction conditions.

### Multiobjective optimisation

To enable extraction of mechanistic insight into solvent-dependent effects through interpretable model parameters using ALaBO, we adopted a two-stage hybrid optimisation *via* Pareto scalarisation strategy. The TSEMO algorithm was first employed to explore continuous variables (concentration, equivalents, residence time and temperature), aiming to maximise the yield of product 3 and minimise the amount of the undesired impurity 4.^30,34^ From this, a Pareto front would be generated which would allow the derivation of a suitable weighted objective function, to simultaneously assess yield and selectivity within a single function. The ALaBO algorithm would then be implemented to perform a mixed variable optimisation including the choice of solvent.^17^ Future work will focus on expanding the capabilities of the ALaBO algorithm such that it will be able to directly handle mixed variable multiobjective problems.^35,36^

**Fig. 3:**
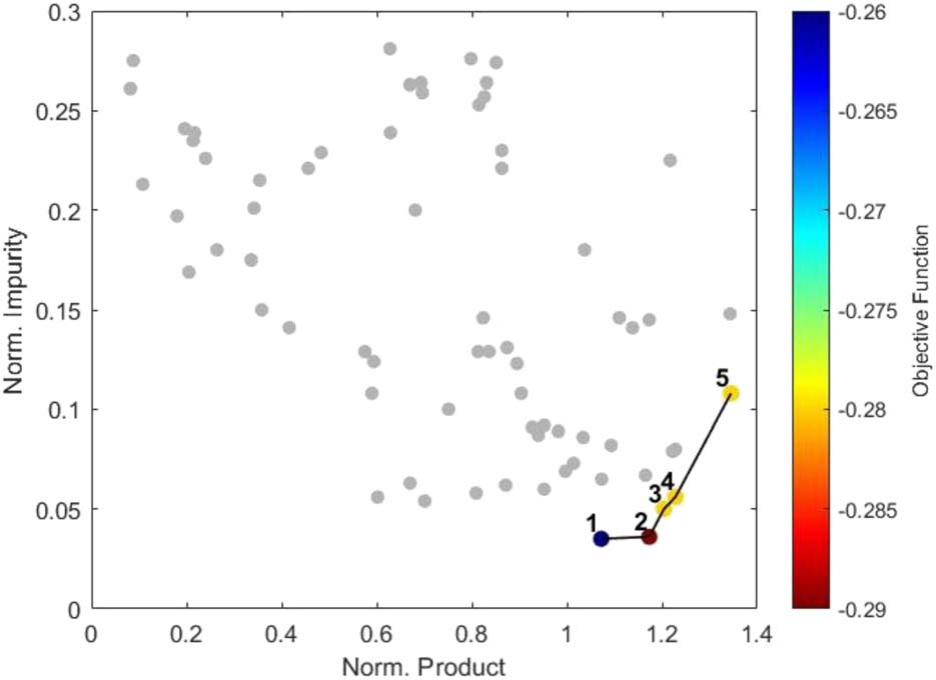
Normalised product, 3, against normalised impurity, 4, from the TSEMO optimisation displaying the Pareto front.

The TSEMO optimisation was carried out using MeCN as the solvent due to its low cost and high availability, in keeping with the other reagents. This allowed the optimisation to be run overnight, without an automated stopping criterion, thus mitigating the risk of premature termination. The optimisation was initialised using a Latin hypercube (LHC) with a sample size of nine, following the 2*n* + 1 rule which has been previously implemented, to create the surrogate models.^37^ A further 60 experiments, 69 in total, were performed and successfully mapped the Pareto front, demonstrating the trade-off between the two outputs (Fig. 3). This is exemplified by point “1”, where 64% conversion and a selectivity ratio of 30.4 are achieved, and point “5”, where an improved conversion of 83% is possible but is accompanied by a decreased selectivity ratio of 12.5.

The Pareto front was analysed to identify the point of diminishing returns *i.e.*, a point where improvement in one objective comes at a disproportionately high cost in the other. Calculating the percentage change in both objectives along the ordered Pareto front showed that moving from point “1” to point “2” gave a 9.4% increase in product formation with only a 2.9% increase in impurity. However, moving from point “2” to point “3” gave a 2.6% increase in product with a 39% increase in impurity. Hence, point “2” was identified as the preferred solution to the optimisation as defined by our criteria, equating to 240 mM of 1, 1 equivalent of 2, 8.71 min and 46 °C to deliver a 56% yield. Although 69 experiments were performed, the Pareto front was mapped after 41 experiments and the preferred solution found after just 11 experiments.

### Mixed variable optimisation

Weightings for the objective function (eqn (1)) were inferred using a reverse engineering approach, by solving a linear program that ensures the selected preferred solution (Fig. 3, point “2”) achieves a minimum value relative to all other points on the Pareto front. Importantly, the objective function remains sensitive to improvements beyond the originally selected point: any new solution offering better yield and/or selectivity will naturally result in an improved value, preserving the flexibility and relevance of the original multiobjective analysis. The colour grading in Fig. 3 demonstrates how well each of the solutions on the Pareto front, from the TSEMO optimisation, satisfy the objective function.

1 *f*(*x*) = −0.2667 × amide(*x*) + 0.773 × enamine(*x*)

**Fig. 4:**
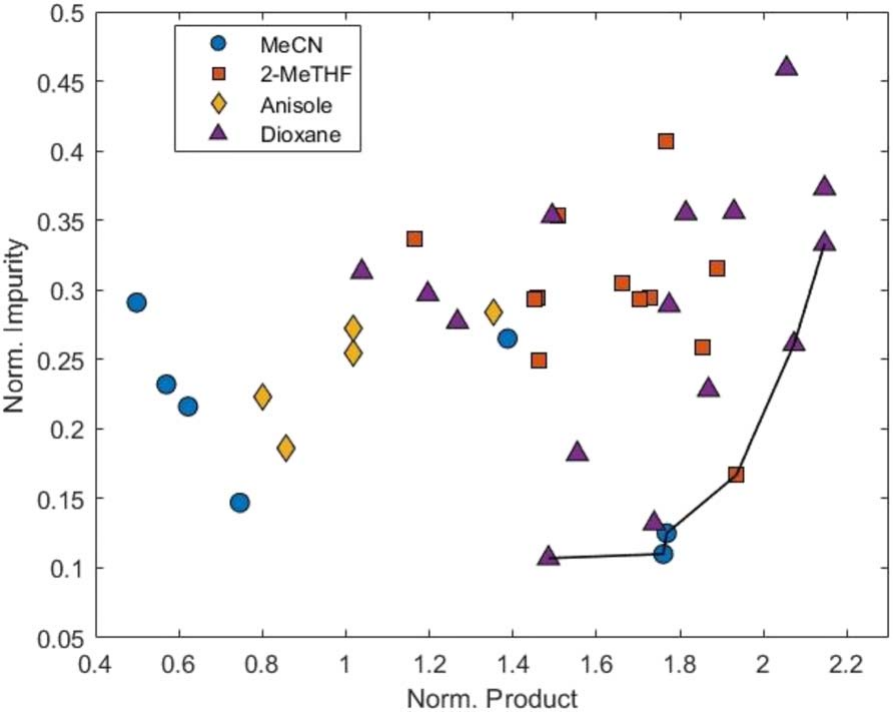
Normalised product, 3, against normalised impurity, 4, from the mixed variable ALaBO optimisation, displaying the Pareto front.

The ALaBO optimisation was initialised using a LHC with a sample size of 12 for the continuous variables.^17^ To ensure adequate representation across categorical levels, the 12 experiments were distributed evenly across the four solvents, resulting in three unique experiments per solvent. As previously, the optimisation was allowed to run overnight to prevent premature termination. A further 27 experiments, 39 in total, were performed until the reaction space had been thoroughly covered and the probability of further improving the objective function was deemed minimal. As the solvent stability study suggested (Fig. 2), there were distinct differences between the results obtained for each solvent (Fig. 4).

It was observed that dioxane facilitated the greatest product formation, but this was widely accompanied by significant impurity formation. In fact, dioxane achieved 100% conversion, but this was associated with a low selectivity ratio of 5.76. Conversely, MeCN was effective at minimising impurity formation, however struggled to deliver large quantities of desired product, demonstrated by MeCN achieving the highest selectivity ratio of 16.0 but with a reduced conversion of 90%. 2-MeTHF displays the best compromise between the high conversion observed in dioxane and the high selectivity observed in MeCN, delivering our optimal solution at 209 mM of 1, 1.7 equivalents of 2, 8.4 min and 30 °C to achieve a 94% yield. This demonstrates a significant increase in yield compared to the preferred solution identified during the first optimisation where the yield was 56%. It is important to note that the lower impurity levels observed in the first optimisation compared to the second reflect the different optimisation strategies. TSEMO explicitly maps the full Pareto front, including regions of low impurity at the expense of product, whereas ALaBO's scalarised single objective formulation inherently prioritises trade-offs that maximise the weighted objective.

**Fig. 5:**
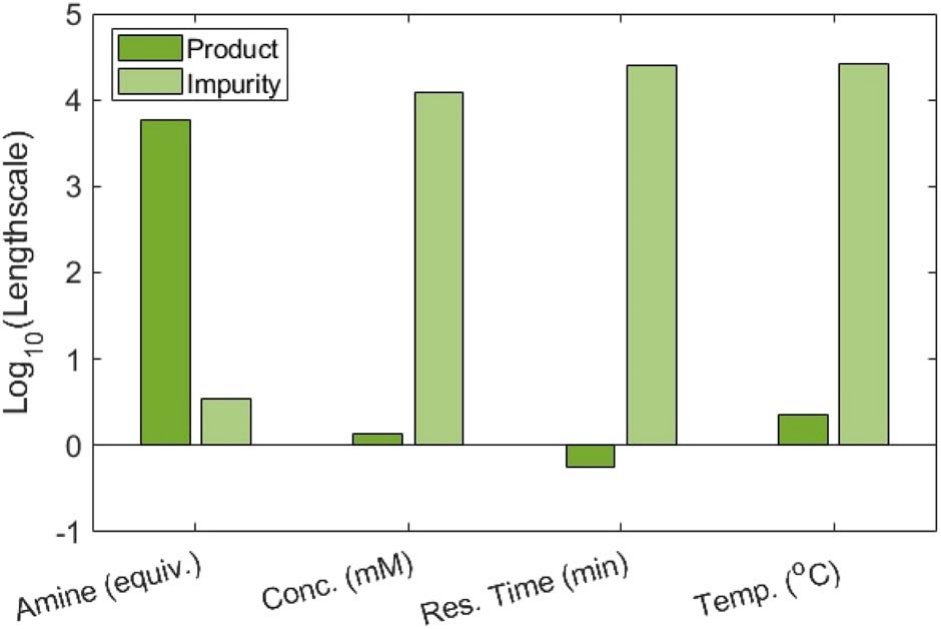
Bar charts representing the kernel lengthscales for each continuous variable with respect to product 3 and impurity 4, plotted on a log_10_ scale.

To better understand the driving factors behind reaction performance, the relative influence of each variable was assessed using the kernel lengthscales from individual GP models of product 3 and impurity 4 formation. Kernel lengthscales are extracted from the correlation function and control how sensitive the model is to changes in each input variable.^38^ For this, normalised peak areas were used rather than concentrations, in keeping with the input to the algorithm. These models were constructed using the same fitting approach as ALaBO. Both showed strong predictive performance, achieving *R*^2^ values of 0.810 and 0.902 respectively during leave-one-out cross-validation (see SI). The kernel lengthscales of each continuous variable were compared to determine their relative importance, where the smaller the value, the greater the level of influence on the output (Fig. 5).

For the formation of product 3, the residence time had the greatest influence, followed by concentration and then temperature, while the amine equivalents played a minor role. The lack of sensitivity of product formation to amine equivalents is consistent with a mechanism in which the enzyme binds the ester substrate and reacts with amine in a subsequent, non-rate-limiting step. In contrast, for formation of impurity 4, the opposite is observed where amine equivalents dominate and residence time, concentration, and temperature have negligible effects. This contrast highlights a divergence in the sensitivity of product and impurity formation to reaction conditions, due to the different reaction mechanisms.

The relationship between solvents can be inferred from the 2D latent variable embeddings produced by the GP models (Table 2). Latent variable embeddings are learned continuous representations of categorical inputs in a low-dimensional space, where each category is assigned coordinates optimised jointly with the model.^39^ In this case, both GPs effectively collapsed the solvent latent space to 1D, indicating that a single latent axis captures all meaningful variation across solvents. For the formation of product 3, dioxane and 2-MeTHF are positioned closely in the latent space, suggesting that they have similar effects on yield. Similarly, anisole and MeCN cluster together, indicating a comparable influence on product formation distinct from that of dioxane and 2-MeTHF. In contrast, the impurity model embeds all four solvents in close proximity, indicating a lower solvent-driven variation. The stronger effect of solvent choice on the formation of product 3 than impurity 4 suggests that the enzyme-catalysed transformation is more sensitive to the reaction environment than the direct nucleophilic addition of amine to ketone. Indeed, the higher yields observed in dioxane and 2-MeTHF, both polar aprotic ether-based solvents, may reflect their coordinating properties, which could support favourable stabilisation of key catalytic intermediates.

**Table 2:** 2D latent variables for each solvent from the GP models of product and impurity

Solvent	Product	Impurity
MeCN	0, 0	0, 0
2-MeTHF	0.870, 0	−0.0044, 0
Anisole	0.173, −8.832 × 10^−9^	−0.0160, −1.928 × 10^−7^
Dioxane	0.932, 1.29 × 10^−8^	−0.0249, −2.894 × 10^−7^

**Fig. 6:**
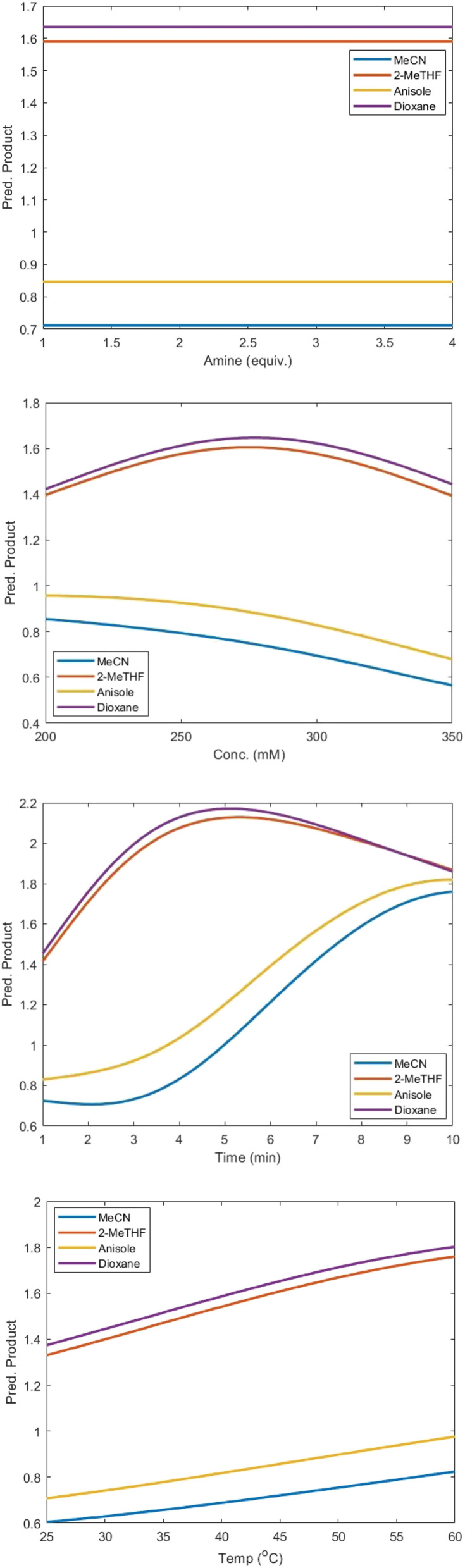
Partial dependence plots showing the effect of each continuous reaction variable on predicted formation of product 3, for each solvent.

To further understand the effects of the four solvents, partial dependence plots (PDPs) were created using predictions from the GP models, by varying each continuous variable while holding the others at their median values (see Fig. 6 for product 3 formation and Fig. S11 for impurity 4 formation). This enabled visualisation of how solvent choice modulates the relationship between individual factors and reaction outcomes, providing a more detailed view of solvent-dependent behaviour.

In agreement with the latent variable embeddings, dioxane and 2-MeTHF showed similar behaviour to each other, but distinct behaviour from anisole and MeCN, across all parameter profiles for the formation of product 3. Furthermore, in agreement with the kernel lengthscales, varying the amine equivalents across all solvents had virtually no impact on product 3. Similarly, the PDPs for the formation of impurity 4 were also in agreement with the kernel lengthscale and latent variable analysis, where varying time, concentration and temperature had virtually no impact, and increasing equivalents of amine resulted in a linear increase in impurity levels, independent of solvent. This linear increase is consistent with a nucleophilic addition mechanism with a bimolecular rate-determining step.

Notably, two distinct process sensitivities were identified for yield across different solvents: (i) increasing time in dioxane and 2-MeTHF increased yield up to ∼5 min, where any further increase resulted in a decrease in yield. In contrast, increasing time in anisole and MeCN always increased yield in a sigmoidal relationship; (ii) increasing concentration in dioxane and 2-MeTHF increased yield up to ∼280 mM, where any further increase resulted in a decrease in yield. In contrast, increasing concentration in anisole and MeCN always resulted in a decrease in yield. Based on our analysis, the decrease in yield observed at higher concentrations and longer reaction times in dioxane and 2-MeTHF is unlikely due to enzyme deactivation or increased impurity formation. Instead, it may arise from product inhibition effects that become more pronounced at higher conversions, highlighting the complex interplay between reaction conditions and biocatalytic efficiency. These findings demonstrate the advantage of this optimisation approach, as it enables the discovery of non-intuitive, functionally relevant relationships between variables and outcomes that may not be evident from traditional chemical reasoning or similarity metrics alone, an obstacle that has historically hindered the broader development of biocatalytic processes.

Overall, the application of this methodology has allowed for 108 experiments to be performed in approximately 31 hours (approximately 3.5 experiments per hour). This has enabled a highly efficient, sustainable, direct synthesis of *N*-benzyl acetoacetamide to be established. Although not included as an objective within the optimisations, our optimised continuous flow process achieved a STY of >270 g L^−1^ h^−1^ (Table 1, entry 7). This demonstrates 270-fold greater productivity compared to previous biocatalytic synthesis (entry 1), as well as enabling the replacement of hazardous and environmentally unfriendly solvent, dioxane, with a safer and greener alternative, 2-MeTHF. Furthermore, this work has achieved 2.5-fold greater STY compared to the most efficient chemical synthesis (entry 4), where stoichiometric amounts of expensive [BMIm]OH ionic liquids were employed.^40^

## Conclusions

This work demonstrates the first implementation of automated BO to perform a multiobjective optimisation of a biocatalytic reaction system, handling both continuous and categorical variables. This methodology has allowed for full optimisation of the reaction in less than 1.5 days (31 h), achieving 94% yield and a STY of >270 g L^−1^ h^−1^. This represents a 270-fold greater STY than previous enzymatic synthesis and 2.5-fold greater STY than previous chemical transformations.^24,27^

The BO approach not only improved the yield and productivity of the reaction, but enabled non-intuitive relationships between reaction parameters to be elucidated. These insights allow greater understanding of interactions between continuous variables, as well as the solvent effects at play within the reaction. This further highlights the advantage of data-driven strategies over traditional reaction optimisations.

The impurity levels were also minimised as part of the optimisation process, giving <5% of the undesired enamine by-product at the optimised conditions. The high purity of the β-ketoamide product stream provides an excellent opportunity for further functionalisation of the highly functionalised API building block without additional work-up and purification. Telescoped chemoenzymatic cascades and their autonomous optimisation are currently under development in our group, which will allow for rapid diversification of chemical space to generate bioactive molecules.

## Author contributions

M. J. T. performed all experimental work, analysed the data, and wrote the first version of the manuscript. S. C. C. conceptualised the ideas. A. D. C. conceptualised the ideas and analysed the data. All authors performed subsequent revisions of the manuscript.

## Conflicts of interest

There are no conflicts to declare.

## Supplementary Material

SC-OLF-D5SC04249F-s001

## Data Availability

The data that support the findings of this study are available in the SI of this article. The code for the algorithms used is available on the following GitHub repositories: TSEMO: https://github.com/Eric-Bradford/TS-EMO, ALaBO: https://github.com/adamc1994/ALaBO. Supplementary information is available. See DOI: https://doi.org/10.1039/d5sc04249f.
