# Supplementary material for: Autonomous optimisation of biocatalytic reactions: enzymatic synthesis of N-benzyl acetoacetamide in continuous flow
Source: Chem Sci. 2025 Sep 10;16(40):18783–90. doi: 10.1039/d5sc04249f (PMC12434490; doi:10.1039/d5sc04249f)
Supplement: SC-016-D5SC04249F-s001 [file SC-016-D5SC04249F-s001.pdf]

Supplementary Information (SI) for Chemical Science.
This journal is © The Royal Society of Chemistry 2025

Autonomous Optimisation of Biocatalytic Reactions: Enzymatic Synthesis of *N*-benzyl acetoacetamide in Continuous Flow

Matthew J. Takle,[a] Sebastian C. Cosgrove[b] and Adam D. Clayton*[a]

[a] *Institute of Process Research and Development, School of Chemistry & School of Chemical Process Engineering, University of Leeds, Leeds, LS2 9JT, UK. E-mail: A.D.Clayton@leeds.ac.uk*
[b] *School of Chemical and Physical Sciences & Centre for Glycoscience, Keele University, Keele, ST5 5BG, UK.*

Supporting Information

1. Materials and Characterisation

Unless otherwise stated, materials were purchased from commercial sources and used as received. ^{1}H and ^{13}C NMR spectra were recorded on a 400 MHz Bruker AVANCE III HD spectrometer. Unless otherwise stated, ^{1}H and ^{13}C NMR chemical shifts are reported in $CDCl_3$, relative to residual protic solvent peaks: δH = 7.26 ppm and δC = 77 ppm, respectively. Multiplicity is denoted as follows: s = singlet, b = broad, d = doublet, t = triplet, q = quartet, p = pentet, m = multiplet and coupling constants (J values) are given in Hz. Reverse phase uHPLC analysis was performed using an Agilent 1290 Infinity II instrument, fitted with an Agilent Poroshell 120 EC-C18 column (100 mm x 2.1 mm x 1.9 µm) using a solvent system of 0.1% formic acid in H_2O (A) and 0.1% formic acid in MeCN (B). The method used was 0 – 1 min (5 % B), 1 – 3 min (5 – 95 % B), 3 – 4 min (95 % B), 4 – 4.5 min (95 – 5 % B) at 0.8 mL min^{-1}. Chromatograms were recorded by a diode array UV-Vis detector, using 254 nm wavelength for quantification.

$$Eq.S1. \quad Normalised\ Product = \frac{Product\ Peak\ Area}{Int.Std.Peak\ Area}$$

$$Eq.S2. \quad Normalised\ Impurity = \frac{Enamine\ Peak\ Area}{Int.Std.Peak\ Area}$$

$$Eq.S3. \quad Conversion\ (\%) = \frac{[Ester]_0 - [Amide]_t}{[Ester]_0} \times 100$$

$$Eq.S4. \quad Yield\ (\%) = \frac{[Amide]_t}{[Ester]_0} \times 100$$

$$Eq.S5. \quad STY = \frac{Mass\ of\ Amide}{Volume\ of\ Reactor\ \times\ Reaction\ Time}$$

2. Automated Continuous Flow Reactor

Volumetric flasks were charged with stock solutions of methyl acetoacetate (1.2 M) and benzyl amine (2 M), alongside a dilution solvent. The inlet lines, PFA tubing (1/16" OD), connected each set of stock solutions to a 16-port multiposition valve (Knauer Azura VU 4.1) which were then connected to three dual piston JASCO HPLC pumps (2 x PU4580, 1 x PU4185) *via* a further length of PFA tubing (1/16" OD). Using stainless steel tubing (1/16" OD), the outlet of each pump was connected to a Swagelok cross union (SS-100-4). The combined reagents were then passed along stainless steel tubing (1/16" OD) to the stainless steel PBR (1/8" OD). The PBR was housed within an aluminium heating jacket, controlled by a PID controller (Eurotherm 3200), and the temperature monitored using a K-type thermocouple. Stainless steel tubing (1/16" OD) connected the PBR to the inlet of a sampling valve (Vici-C84U, 0.05 µL injection volume). The outlet of the sampling valve connected to an Upchurch BPR, manually adjusted to 8 bar, using PFA tubing (1/8" OD). The sampling valve was connected to an Agilent 1290 Infinity II instrument for inline analysis of the reaction. The multiposition valve (USB A), pumps (RS232) and PID controller (RS232) were all connected directly to a PC and controlled using MATLAB code. The sample valve was connected directly to the HPLC and used as an external valve in the ChemStation software.

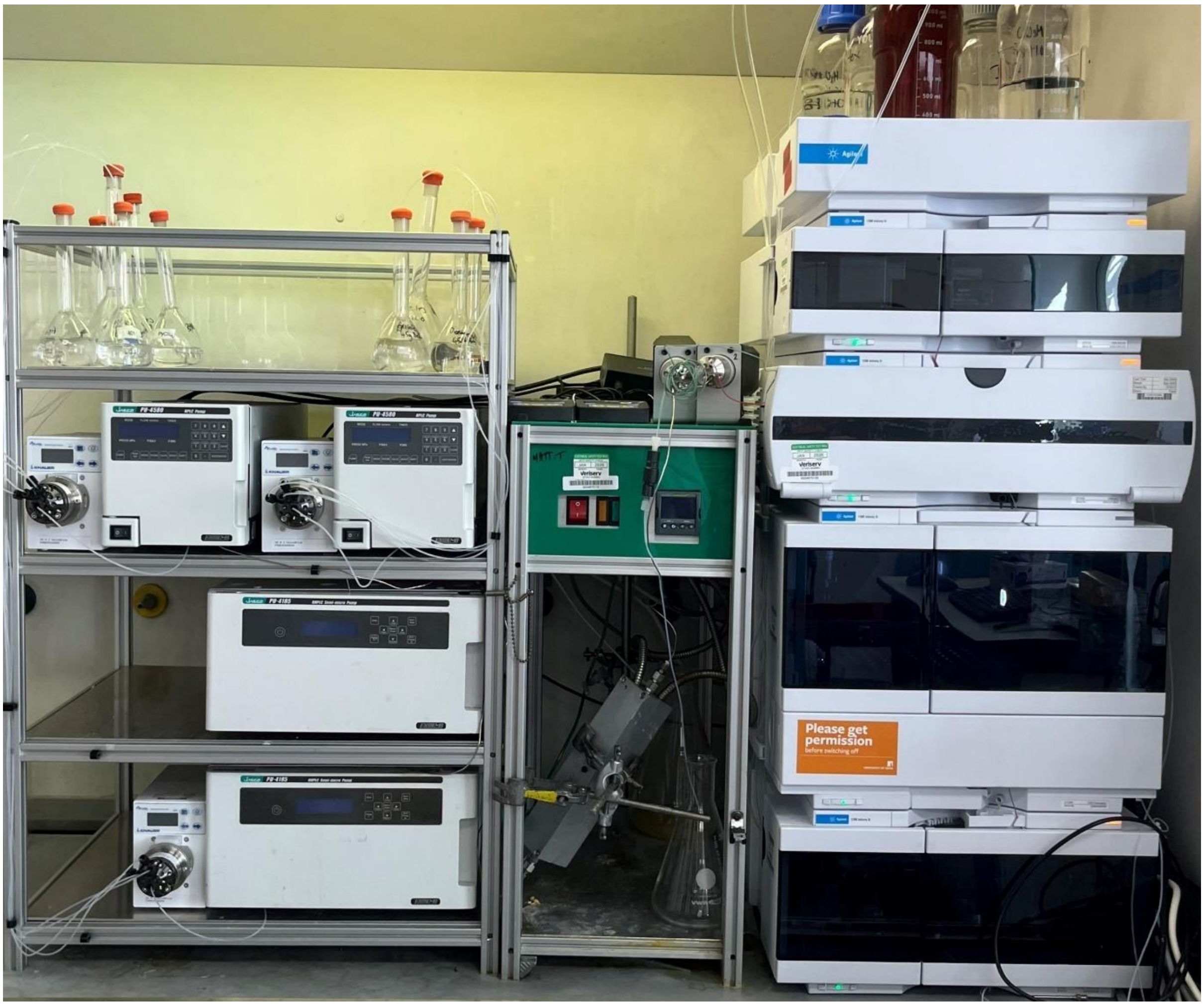

Fig. S1. Picture of automated flow reactor system.

3. Packed Bed Reactor Preparation

Stainless steel tubing (1/8” OD) was capped at both ends with metal frits and sealed using Swagelok fittings. The column was packed with Novozym-435 (988 mg) and washed with MeCN (50 mL) to remove any leachate. The volume of the PBR volume was calculated by taking the difference between dry and wet weights of the column, to give a void volume of 2.25 mL (MeCN density: 0.78 g cm^{-3} at 20 °C).[1] Fresh packed bed reactors were washed with MeCN at 0.5 mL min^{-1} until on-line HPLC showed that no leachate was remaining.

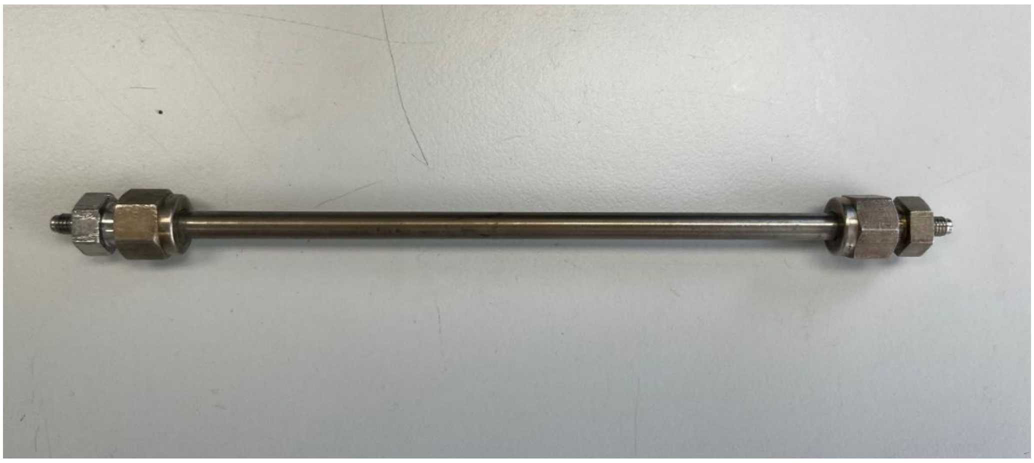

Fig. S2. Picture of Novozym-435 packed bed reactor.

4. Typical procedure for the synthesis of *N*-benzyl acetoacetamide in flow, 3.

The flow reactor and packed bed reactor were used as described in S2. and S3. Each pump was primed *via* the priming port, then all pumps were set to 0.5 mL min^{-1} to saturate the system for 10 minutes and the Eurotherm set to 30 °C. The flow rate of each pump was then reduced to 0.1 mL min^{-1} ("dead-time" conditions) prior to starting the optimisation to save material. The flow reactor was controlled from the PC using MATLAB code previously established in the group.[2] The target temperature was set, and once reached each individual pump was set to the desired flow rate. Once steady state was achieved, an online HPLC sample was taken using the sampling valve. The temperature and flow rates returned to the "dead-time" conditions, awaiting the next target conditions to be set.

5. TSEMO Optimisation

5.1. Optimisation Parameters

Table S1. Table of TSEMO optimisation input upper/lower bounds and optimisation objectives.

Reaction Parameter	Lower Bound	Upper Bound
Amine (equiv.)	1	4
Conc. (mM)	200	350
Res. Time (min)	1	10
Temp (°C)	25	60
**Objective**	Max. Norm. Product	Min. Norm. Impurity

5.2. Workflow

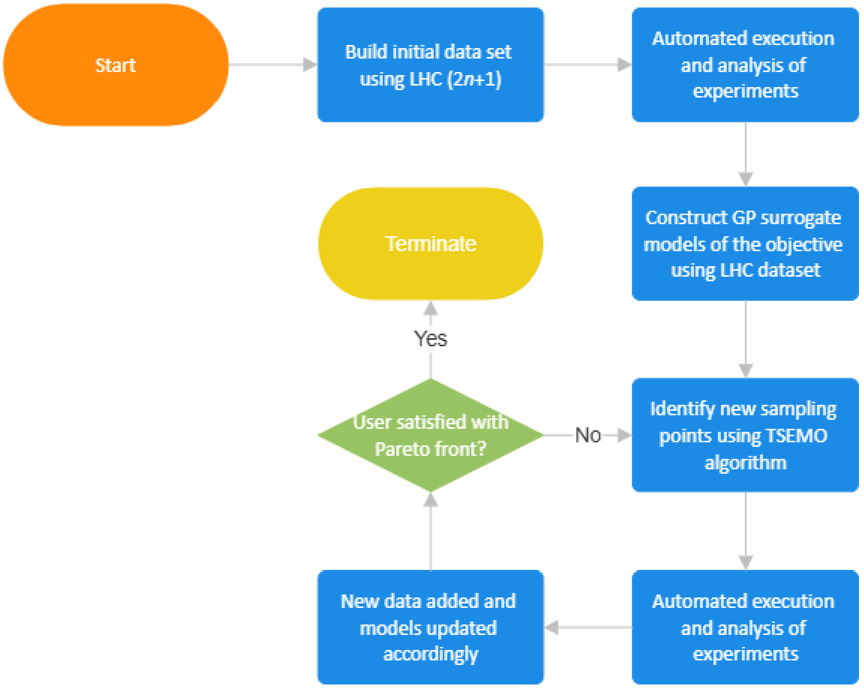

Fig. S3. Flow chart for the TSEMO optimisation. Further detail available of TSEMO algorithm see Clayton et al, 2020.[2]

6. ALaBO Optimisation

6.1. Optimisation Parameters

Table S2. Table of ALaBO optimisation input upper/lower bounds and optimisation objectives.

Reaction Parameter	Lower Bound		Upper Bound	
Amine (equiv.)	1		4	
Conc. (mM)	200		350	
Res. Time (min)	1		10	
Temp (°C)	25		60	
Solvent	MeCN	2-MeTHF	Anisole	Dioxane
**Objective**	Minimise $f(x)$			

$$Eq.S5 \quad f(x) = -0.2667 \cdot Amide(x) + 0.773 \cdot Enamine(x)$$

6.2. Workflow

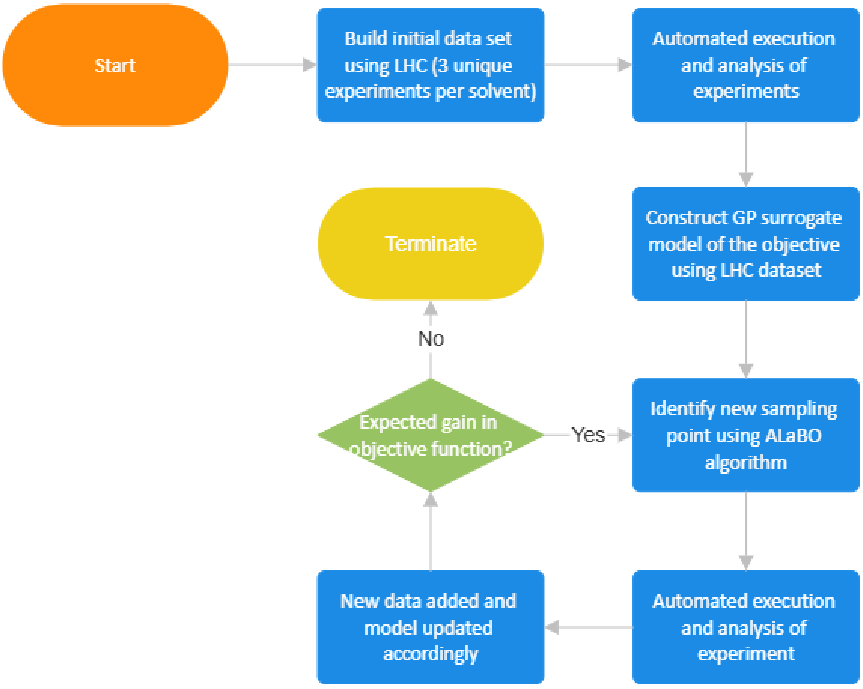

Fig. S4. Flow chart for the ALaBO optimisation. Further detail available of ALaBO algorithm see Aldulaijan et al, 2024.[3]

7. Reaction Datasets

7.1. Solvent Stability Experiment

Table S3. Dataset from the solvent stability study of Novozym-435 monitoring *N*-benzyl acetoacetamide, **3**.

Exp	Time (h)	Solvent	Amide (mM)	Exp	Time (h)	Solvent	Amide (mM)
1	0.25	MeCN	320	49	12.25	MeCN	325
2	0.5	MeCN	271	50	12.5	MeCN	274
3	0.75	MeCN	227	51	12.75	MeCN	256
4	1	MeCN	212	52	13	MeCN	237
5	1.25	MeCN	209	53	13.25	MeCN	222
6	1.5	MeCN	204	54	13.5	MeCN	218
7	1.75	MeCN	206	55	13.75	MeCN	211
8	2	MeCN	206	56	14	MeCN	212
9	2.25	MeCN	205	57	14.25	MeCN	210
10	2.5	MeCN	205	58	14.5	MeCN	209
11	2.75	MeCN	206	59	14.75	MeCN	207
12	3	MeCN	206	60	15	MeCN	208
13	3.25	2-MeTHF	205	61	15.25	2-MeTHF	205
14	3.5	2-MeTHF	190	62	15.5	2-MeTHF	190
15	3.75	2-MeTHF	206	63	15.75	2-MeTHF	218
16	4	2-MeTHF	209	64	16	2-MeTHF	222
17	4.25	2-MeTHF	212	65	16.25	2-MeTHF	225
18	4.5	2-MeTHF	217	66	16.5	2-MeTHF	227
19	4.75	2-MeTHF	216	67	16.75	2-MeTHF	224
20	5	2-MeTHF	217	68	17	2-MeTHF	227
21	5.25	2-MeTHF	219	69	17.25	2-MeTHF	227
22	5.5	2-MeTHF	219	70	17.5	2-MeTHF	227
23	5.75	2-MeTHF	220	71	17.75	2-MeTHF	228
24	6	2-MeTHF	220	72	18	2-MeTHF	228
25	6.25	Anisole	221	73	18.25	Anisole	228
26	6.5	Anisole	217	74	18.5	Anisole	226
27	6.75	Anisole	138	75	18.75	Anisole	137
28	7	Anisole	135	76	19	Anisole	130
29	7.25	Anisole	135	77	19.25	Anisole	130
30	7.5	Anisole	137	78	19.5	Anisole	133
31	7.75	Anisole	138	79	19.75	Anisole	132
32	8	Anisole	138	80	20	Anisole	129
33	8.25	Anisole	139	81	20.25	Anisole	131
34	8.5	Anisole	141	82	20.5	Anisole	130
35	8.75	Anisole	140	83	20.75	Anisole	130
36	9	Anisole	140	84	21	Anisole	131
37	9.25	Dioxane	141	85	21.25	Dioxane	129
38	9.5	Dioxane	178	86	21.5	Dioxane	148
39	9.75	Dioxane	315	87	21.75	Dioxane	301
40	10	Dioxane	316	88	22	Dioxane	307

41	10.25	Dioxane	317	89	22.25	Dioxane	307
42	10.5	Dioxane	317	90	22.5	Dioxane	312
43	10.75	Dioxane	318	91	22.75	Dioxane	311
44	11	Dioxane	319	92	23	Dioxane	312
45	11.25	Dioxane	317	93	23.25	Dioxane	312
46	11.5	Dioxane	317	94	23.5	Dioxane	313
47	11.75	Dioxane	319	95	23.75	Dioxane	312
48	12	Dioxane	318	96	24	Dioxane	312

7.2. TSEMO Optimisation

Table S4. Dataset from the TSEMO self-optimisation of the synthesis of *N*-benzyl acetoacetamide, **3**. Preferred solution highlighted in green.

Exp	Amine (Equiv.)	Sol. Conc. (mM)	Res. Time (min)	Temp (°C)	Norm. Prod.	Norm. Imp	Conv (%)	Sel. Ratio	Obj. Func.
1	2.19	349	9.31	57.8	1.343	0.148	82	9.06	-0.24
2	3.10	296	7.57	47.9	1.217	0.225	72	5.40	-0.15
3	1.69	253	6.16	49.6	1.345	0.108	83	12.50	-0.28
4	2.41	316	3.27	53.8	1.037	0.180	57	5.76	-0.14
5	2.77	319	4.64	32.6	0.681	0.200	43	3.40	-0.03
6	3.63	282	5.55	36.4	0.798	0.276	53	2.89	0.00
7	3.87	206	2.61	40.0	0.628	0.281	44	2.24	0.05
8	1.14	242	8.22	28.8	1.073	0.065	69	16.47	-0.24
9	1.51	230	1.27	42.8	0.590	0.108	41	5.45	-0.07
10	1.01	287	4.63	35.6	0.809	0.058	51	13.91	-0.17
11	1.00	240	8.71	46.4	1.173	0.036	75	32.73	-0.29
12	1.00	240	8.60	47.6	1.228	0.056	69	22.06	-0.28
13	1.00	243	7.88	54.2	1.204	0.050	76	24.23	-0.28
14	2.60	240	1.76	28.3	0.264	0.180	27	1.47	0.07
15	1.59	251	8.15	30.4	0.927	0.091	56	10.18	-0.18
16	1.60	252	10.00	31.0	1.014	0.073	61	13.89	-0.21
17	1.59	251	10.00	33.4	1.093	0.082	62	13.36	-0.23
18	2.37	314	4.98	45.5	0.813	0.129	52	6.31	-0.12
19	2.37	304	4.98	45.7	0.836	0.129	59	6.46	-0.12
20	2.37	303	4.62	45.8	0.824	0.146	54	5.66	-0.11
21	2.55	311	6.29	60.0	1.110	0.146	64	7.59	-0.18
22	3.92	295	9.56	40.2	0.831	0.264	57	3.15	-0.02
23	3.93	295	9.55	40.8	0.851	0.274	49	3.10	-0.01
24	3.98	294	9.31	42.5	0.826	0.257	63	3.22	-0.02
25	3.97	294	9.34	42.7	0.815	0.253	58	3.22	-0.02
26	3.26	278	1.00	25.0	0.108	0.213	26	0.51	0.14
27	3.27	277	1.00	25.2	0.082	0.261	17	0.32	0.18
28	3.25	278	1.00	27.0	0.088	0.275	21	0.32	0.19
29	3.22	274	1.00	44.6	0.240	0.226	28	1.06	0.11
30	2.28	259	5.90	32.1	0.594	0.124	51	4.79	-0.06
31	2.32	261	3.15	32.5	0.416	0.141	40	2.95	0.00

32	2.31	261	1.31	32.8	0.205	0.169	25	1.21	0.08
33	2.12	263	2.33	46.4	0.575	0.129	44	4.46	-0.05
34	3.56	350	9.75	28.2	0.456	0.221	42	2.06	0.05
35	3.58	350	9.77	28.3	0.483	0.229	39	2.11	0.05
36	1.81	232	4.07	50.9	0.940	0.087	53	10.79	-0.18
37	1.91	235	4.96	51.1	1.034	0.086	68	12.08	-0.21
38	1.63	238	7.49	36.4	0.871	0.062	63	13.95	-0.18
39	1.63	238	7.56	36.4	0.997	0.069	62	14.48	-0.21
40	1.63	238	7.09	37.3	0.952	0.060	64	16.00	-0.21
41	1.01	350	7.56	45.3	1.072	0.035	64	30.40	-0.26
42	2.25	327	3.20	58.8	0.751	0.100	53	7.55	-0.12
43	2.24	328	5.43	59.5	0.982	0.089	63	11.06	-0.19
44	1.86	346	10.00	59.7	1.228	0.080	76	15.31	-0.27
45	1.87	346	10.00	60.0	1.222	0.079	73	15.51	-0.27
46	3.27	223	1.11	36.3	0.180	0.197	30	0.91	0.10
47	3.27	223	1.29	36.7	0.196	0.241	24	0.81	0.13
48	3.27	224	1.32	38.5	0.217	0.239	26	0.91	0.13
49	3.27	224	1.32	38.5	0.214	0.235	24	0.91	0.12
50	3.12	337	2.33	45.5	0.336	0.175	40	1.93	0.05
51	3.00	350	1.60	51.2	0.342	0.201	35	1.70	0.06
52	3.03	347	1.67	51.5	0.354	0.215	32	1.64	0.07
53	2.13	350	1.33	51.8	0.358	0.150	29	2.38	0.02
54	3.69	266	10.00	44.3	0.863	0.221	60	3.90	-0.21
55	3.70	266	9.50	44.8	0.863	0.230	61	3.75	-0.05
56	2.62	224	8.99	52.5	1.138	0.141	74	8.05	-0.19
57	2.65	221	8.90	54.2	1.173	0.145	73	8.11	-0.20
58	1.83	329	9.46	41.2	0.952	0.092	62	10.37	-0.18
59	1.97	329	7.78	42.4	0.904	0.108	59	8.38	-0.16
60	2.28	335	7.83	44.0	0.874	0.131	58	6.67	-0.13
61	2.19	335	7.83	44.4	0.895	0.123	58	7.25	-0.14
62	1.27	252	7.59	25.0	0.602	0.056	55	10.78	-0.12
63	1.10	253	6.35	25.0	0.701	0.054	45	13.00	-0.15
64	1.17	252	6.25	25.5	0.670	0.063	50	10.68	-0.13
65	1.37	248	7.67	57.3	1.165	0.067	75	17.32	-0.26
66	4.00	320	8.59	41.1	0.629	0.239	58	2.63	0.02
67	4.00	320	8.77	43.1	0.696	0.259	51	2.69	0.01
68	3.93	329	6.70	47.2	0.670	0.263	49	2.55	0.02
69	3.95	330	6.68	48.4	0.693	0.264	50	2.62	0.02

7.3. ALaBO Optimisation

Table S5. Dataset from the ALaBO self-optimisation of the synthesis of *N*-benzyl acetoacetamide, **3**. Preferred solution highlighted in green.

Exp	Amine (Equiv.)	Sol. Conc. (mM)	Res. Time (min)	Temp (°C)	Solvent	Norm. Prod.	Norm. Imp.	Conv (%)	Sel. Ratio	Obj. Func.
1	3.99	223	2.19	57.8	2-MeTHF	1.768	0.407	88	4.34	-0.157
2	2.61	270	7.01	47.9	MeCN	1.387	0.265	73	5.23	-0.165
3	2.78	306	1.52	49.6	Dioxane	1.774	0.289	78	6.14	-0.250
4	2.33	286	2.72	53.8	Anisole	1.017	0.255	74	3.99	-0.074
5	2.20	330	5.75	32.6	Anisole	1.355	0.284	96	4.77	-0.142
6	3.41	322	3.60	36.4	2-MeTHF	1.889	0.316	97	5.99	-0.260
7	1.14	258	9.48	40.0	MeCN	1.760	0.110	88	16.00	-0.384
8	1.26	293	8.75	28.8	MeCN	1.768	0.125	89	14.11	-0.375
9	1.71	209	8.37	29.8	2-MeTHF	1.935	0.167	96	11.61	-0.387
10	1.79	346	4.65	30.8	Anisole	0.857	0.186	88	4.59	-0.084
11	3.59	242	6.25	42.8	Dioxane	2.146	0.373	100	5.76	-0.284
12	3.16	234	5.37	35.6	Dioxane	2.146	0.333	98	6.45	-0.315
13	2.94	350	1.00	46.4	MeCN	0.499	0.291	28	1.71	0.092
14	1.80	324	1.00	47.6	Dioxane	1.554	0.182	72	8.55	-0.274
15	3.00	201	1.00	54.2	Dioxane	1.196	0.297	60	4.02	-0.089
16	3.52	302	2.45	28.3	Dioxane	1.814	0.355	83	5.11	-0.209
17	2.43	298	10.00	30.4	Dioxane	2.073	0.261	97	7.93	-0.351
18	3.38	311	1.00	31.0	2-MeTHF	1.165	0.336	62	3.47	-0.051
19	1.49	309	1.00	33.4	MeCN	0.746	0.147	43	5.07	-0.085
20	2.23	309	1.00	45.5	Anisole	0.800	0.223	52	3.58	-0.041
21	1.00	298	1.21	45.7	Dioxane	1.485	0.107	71	13.90	-0.314
22	1.00	309	10.00	45.8	Dioxane	1.738	0.132	89	13.17	-0.361
23	3.83	200	4.36	42.4	Dioxane	2.055	0.459	90	4.47	-0.193
24	1.88	308	2.77	41.9	Dioxane	1.868	0.228	82	8.20	-0.322
25	2.42	342	1.00	43.6	Dioxane	1.267	0.277	62	4.58	-0.124
26	1.92	328	1.53	39.5	MeCN	0.622	0.216	38	2.88	0.001
27	2.74	265	1.52	48.0	2-MeTHF	1.459	0.294	77	4.96	-0.162
28	2.89	261	1.74	49.1	2-MeTHF	1.507	0.353	71	4.27	-0.129
29	2.95	293	1.51	39.7	Dioxane	1.494	0.353	65	4.23	-0.125
30	2.71	331	1.58	48.2	2-MeTHF	1.451	0.293	71	4.95	-0.160
31	2.11	261	2.43	27.4	MeCN	0.570	0.232	38	2.45	0.028
32	2.63	214	1.51	49.3	Anisole	1.018	0.272	62	3.74	-0.061
33	2.85	282	1.52	49.8	2-MeTHF	1.662	0.305	84	5.44	-0.207
34	2.72	261	1.52	47.8	2-MeTHF	1.728	0.294	84	5.87	-0.233
35	3.03	266	1.71	54.4	Dioxane	1.929	0.356	86	5.42	-0.239
36	2.35	265	1.17	40.8	2-MeTHF	1.463	0.249	76	5.88	-0.198
37	2.72	261	1.52	47.9	2-MeTHF	1.705	0.293	84	5.81	-0.228
38	2.76	271	1.51	48.6	2-MeTHF	1.853	0.259	88	7.16	-0.294
39	2.75	312	1.00	25.7	Dioxane	1.038	0.313	45	3.32	-0.035

8. Post-Optimisation Data Analysis

To evaluate the predictive performance of our Gaussian Process (GP) models for both product and impurity formation, we implemented leave-one-out cross-validation (LOOCV). In this approach, each data point is excluded once from the dataset and used as a test case while the model is trained on the remaining data. The loop iterates over all data points, systematically excluding one at a time, and fits a GP model to the reduced dataset using our the ALaBO framework. The left-out input is then predicted using the trained GP.

8.1. GP Model for *N*-benzyl acetoacetamide, 3

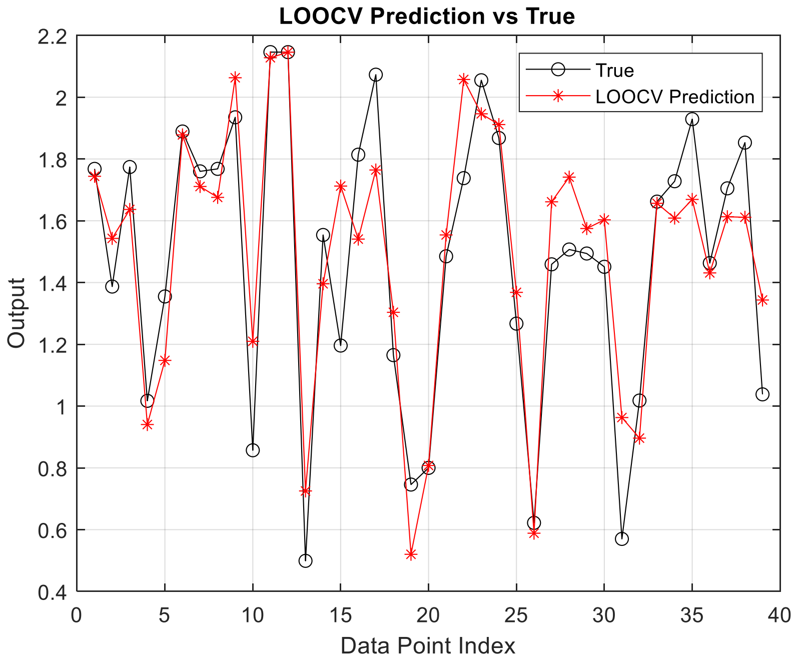

Fig. S5. Output against data point index plot for true values and LOOCV prediction for **3**.

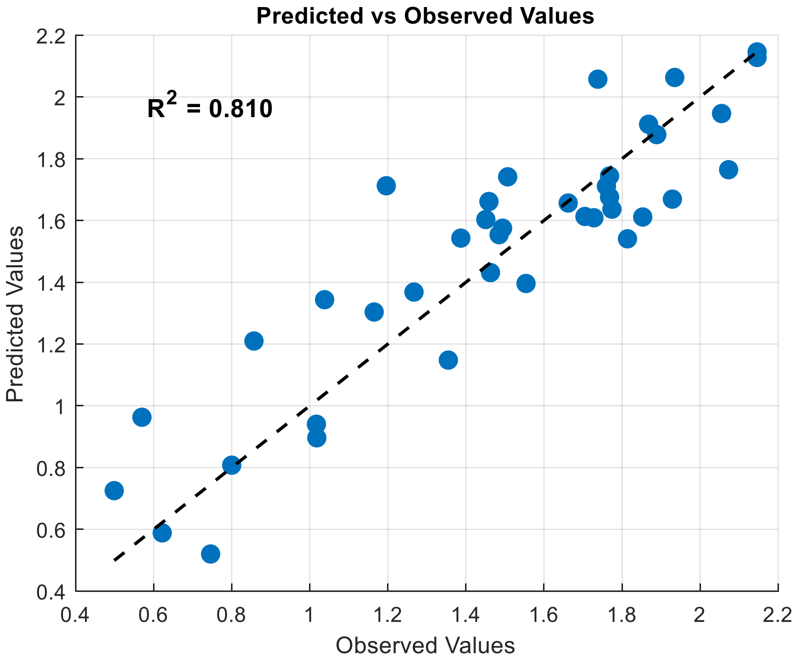

Fig. S6. Predicted vs. observed plot for LOOCV of GP model of **3**.

8.2. Model for Methyl 3-(benzylamino)but-2-enoate, 4

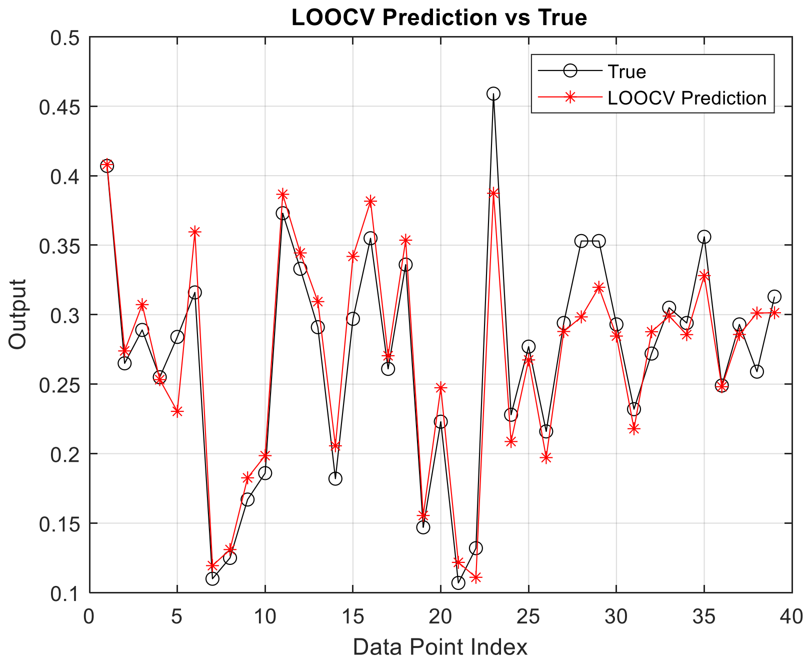

Fig. S7. Output against data point index plot for true values and LOOCV prediction for **4**.

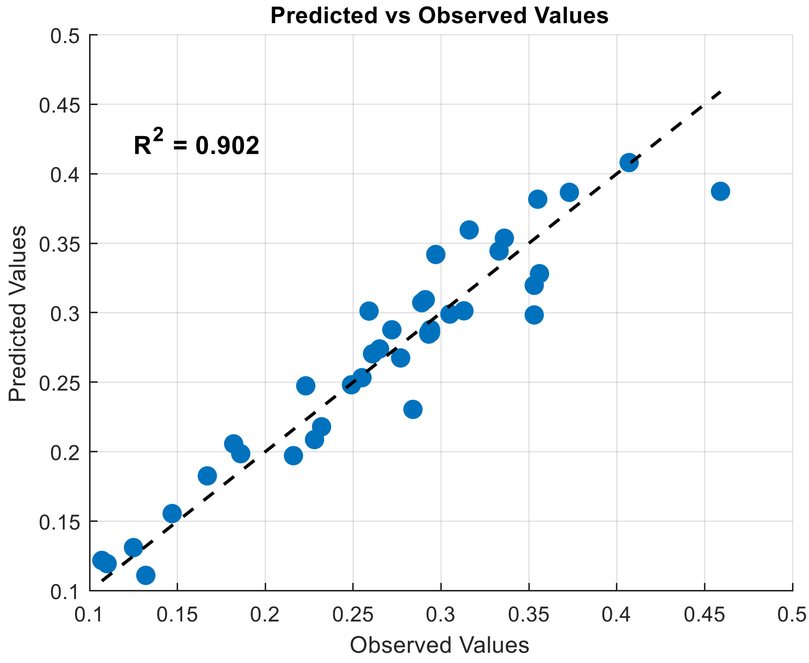

Fig. S8. Predicted vs. observed plot for LOOCV of GP model of **4**.

8.3. Lengthscale Extraction

In the GP models used in this study, the characteristic lengthscales for the quantitative input variables are encoded via log-transformed inverse squared lengthscale parameters, denoted as ϕ (phi). These parameters appear in the correlation function through the term $\mathbf{10^{\phi i}}$, which controls the sensitivity of the GP to changes in each input dimension. To obtain the true lengthscales $\boldsymbol{l_i}$, which represent the effective distance over which the function varies significantly with respect to input dimension $\boldsymbol{i}$, we apply the transformation $\boldsymbol{l_i} = \mathbf{10^{-\phi_{i/2}}}$.

Table S6. Kernel lengthscales for each continuous variables with respect to **3**.

**Factor**	**Kernel Lengthscale**
Time	0.5504
equiv.	5.9755×10^3
conc.	1.3439
Temp	2.2551

Table S7. Kernel lengthscales for each continuous variables with respect to **4**.

**Factor**	**Kernel Lengthscale**
Time	2.4885×10^4
equiv.	3.5433
conc.	1.2198×10^4
Temp	2.5681×10^4

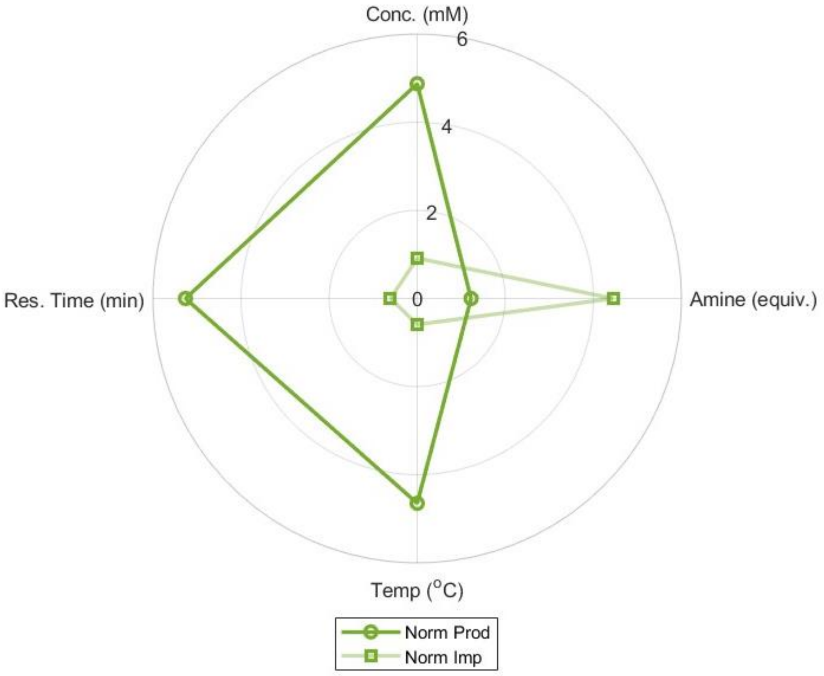

Fig. S9. Radar plot showing the kernel lengthscales for each continuous variable with respect to product and impurity formation, plotted on a Log10 scale. The lengthscales have been inverted for visualisation, such that larger values correspond to greater influence.

8.4. 2D Latent Space

ALaBO handles mixed variables by learning a continuous, 2D latent representation of the categorical inputs. During training, the latent coordinates for the categorical levels are treated as free parameters and optimised via maximum likelihood, jointly with the GP hyperparameters. Hence, the discrete categorical inputs are replaced with their corresponding latent vectors, allowing standard kernel functions to be applied in a unified, continuous input space. The fitted latent embeddings, z, are stored in the GP model and can be extracted post-training for interpretation.[4]

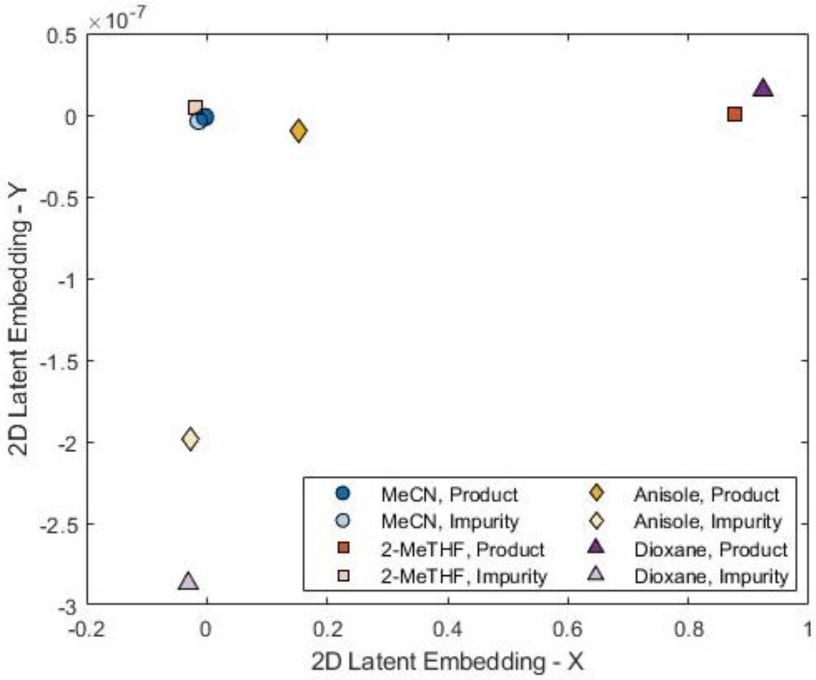

Fig. S10. Scatter plot of the 2D latent embeddings for the formation of product, **3**, and impurity, **4**, under each solvent.

8.5. GP Partial dependence plots for Methyl 3-(benzylamino)but-2-enoate, 4

Partial dependence plots (PDPs) were generated to visualise the effect of individual continuous variables on the predicted response across each solvent. A single continuous variable was varied over a linearly spaced grid of 100 points between its lower and upper bounds as defined in the optimisations, while all other continuous variables were fixed at their median values observed across the explored conditions. The median values were: amine equiv. = 2.71; conc. = 293 mM; time = 1.58 mins; temperature = 43.6 °C.

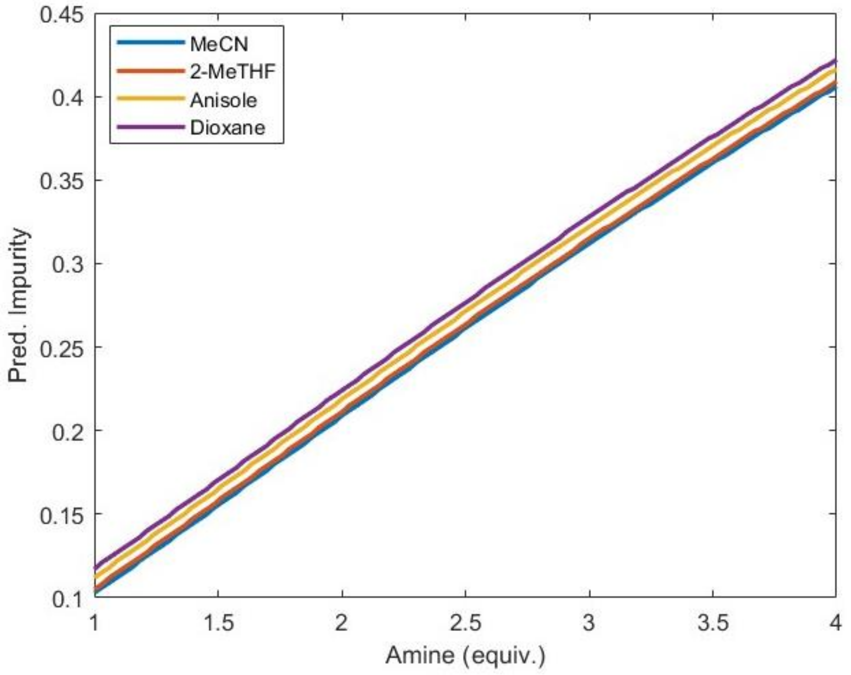
0.45
0.4
0.35
0.3
0.25
0.2
0.15
0.1
Pred. Impurity
1
1.5
2
2.5
3
3.5
4
Amine (equiv.)
MeCN
2-MeTHF
Anisole
Dioxane

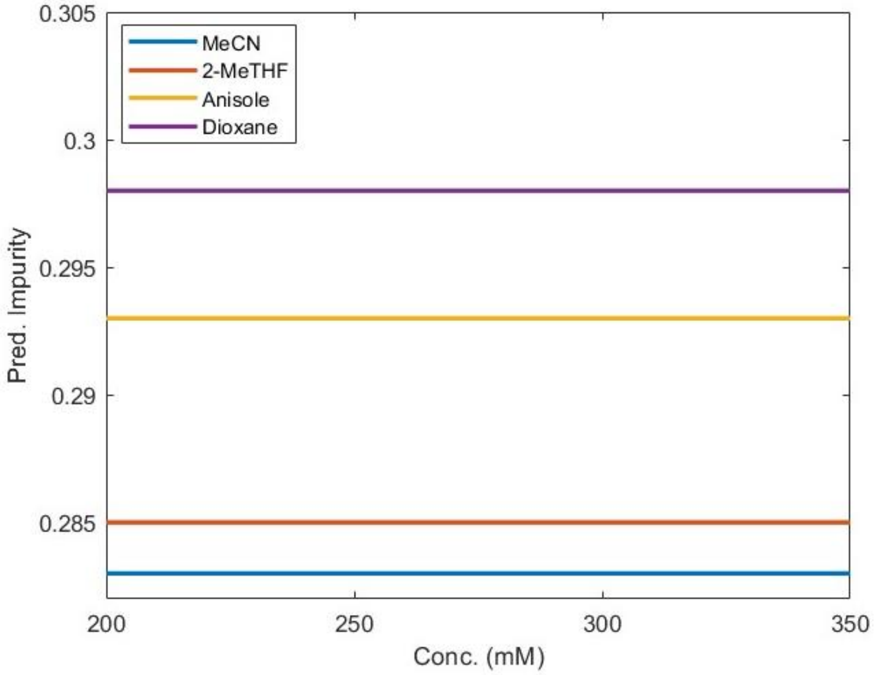
0.305
0.3
0.295
0.29
0.285
Pred. Impurity
200
250
300
350
Conc. (mM)
MeCN
2-MeTHF
Anisole
Dioxane

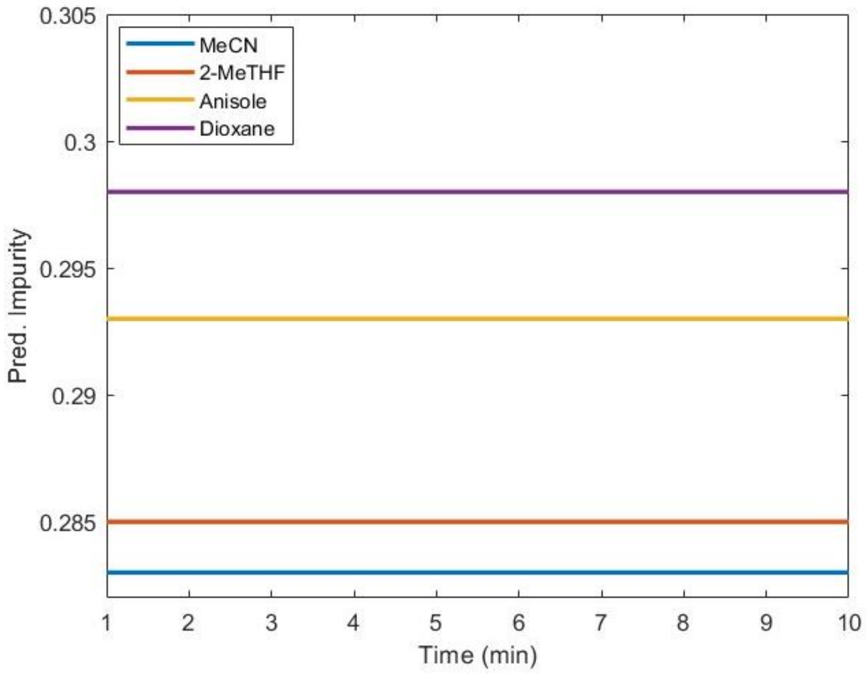

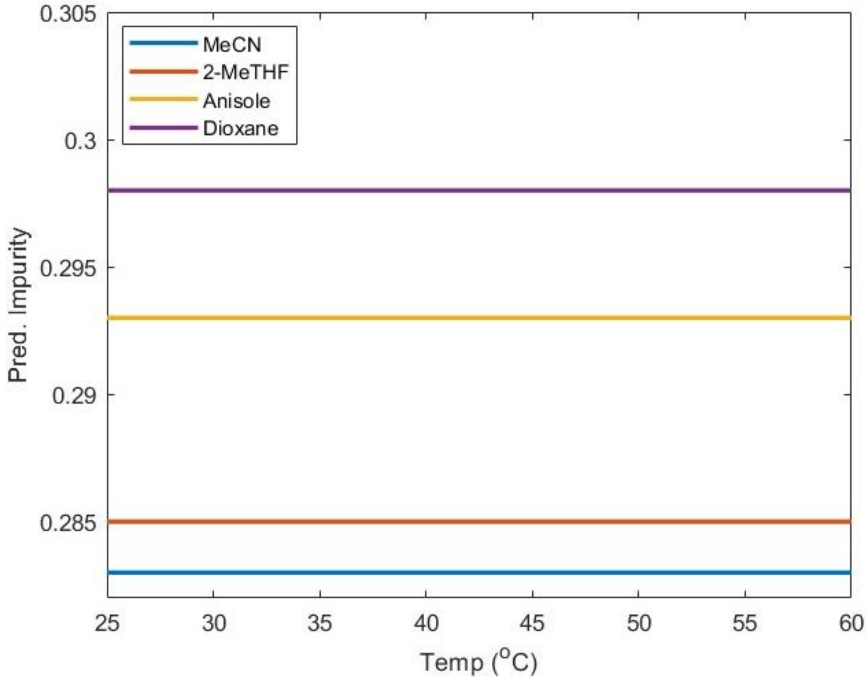

Fig. S11. Partial dependence plots showing the effect of each continuous reaction variable on predicted formation of **4**, for each solvent.

9. NMR Spectra

9.1. *N*-benzyl acetoacetamide, 3

The structure of *N*-benzyl acetoacetamide, **3**, was confirmed through ^{1}H and ^{13}C NMR, alongside LC-MS. The following data was consistent with literature.[5] LC-MS: 191.88 [M+H]. ^{1}H NMR (400 MHz, $CDCl_3$) δ 7.34 – 7.28 (m, 3H), 7.25 – 7.20 (m, 2H), 4.44 (d, *J* = 5.8 Hz, 2H), 3.42 (s, 2H), 2.24 (s, 3H). ^{13}C NMR (101 MHz, $CDCl_3$) δ 204.8, 165.5, 138.0, 128.8, 127.8, 127.7, 49.6, 43.7, 31.2.

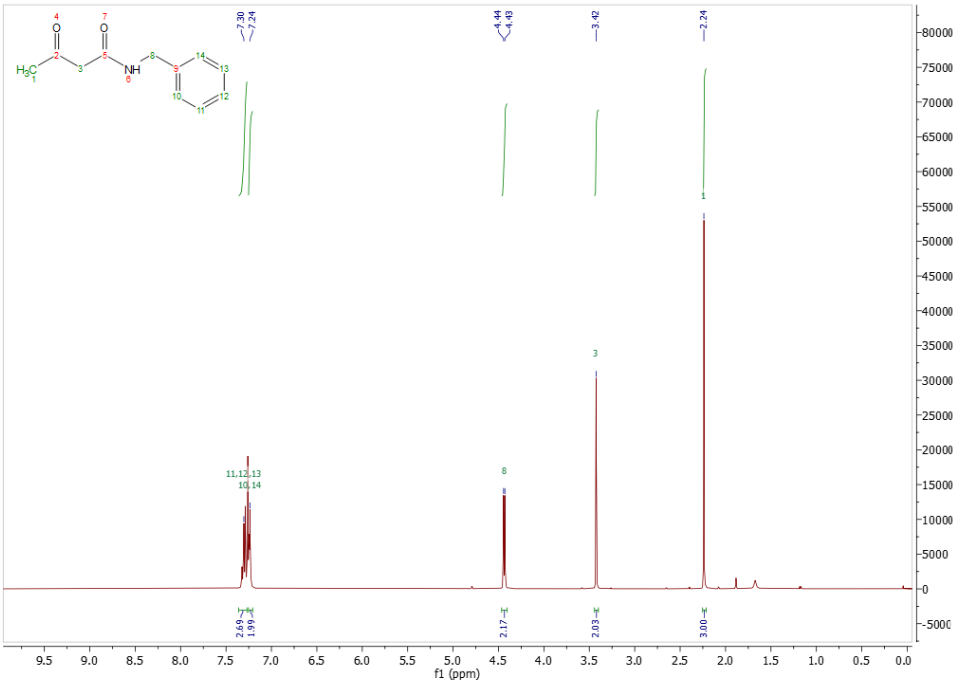

Fig. S12. ^{1}H NMR spectra of *N*-benzyl acetoacetamide, **3**.

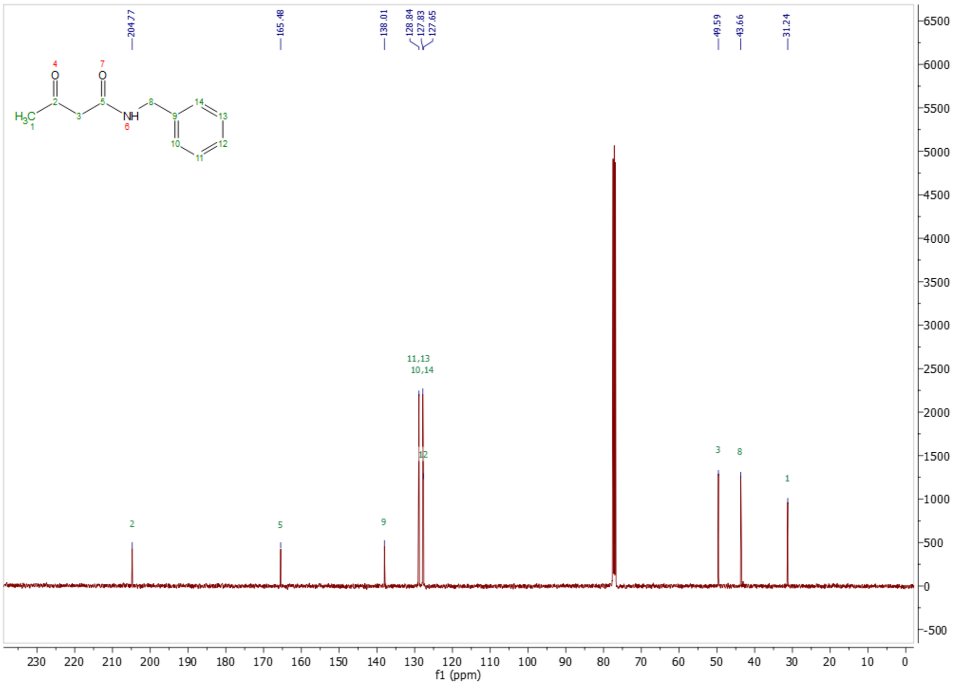

Fig. S13. ^{13}C NMR spectra of N-benzyl acetoacetamide, **3**.

9.2. Methyl 3-(benzylamino)but-2-enoate, 4.

The structure of methyl 3-(benzylamino)but-2-enoate, **4**, was confirmed through ^{1}H and ^{13}C NMR, alongside LC-MS. The following data was consistent with literature.[6] LC-MS: 205.84 [M+H]. ^{1}H NMR (400 MHz, $CDCl_3$) δ 8.94 (s, 1H), 7.36 – 7.30 (m, 2H), 7.28 – 7.21 (m, 3H), 4.54 (s, 1H), 4.41 (d, *J* = 6.4 Hz, 2H), 3.62 (s, 3H), 1.90 (s, 3H). ^{13}C NMR (101 MHz, $CDCl_3$) δ 170.9, 162.0, 138.7, 128.8, 127.4, 126.7, 82.8, 50.0, 46.8, 19.4.

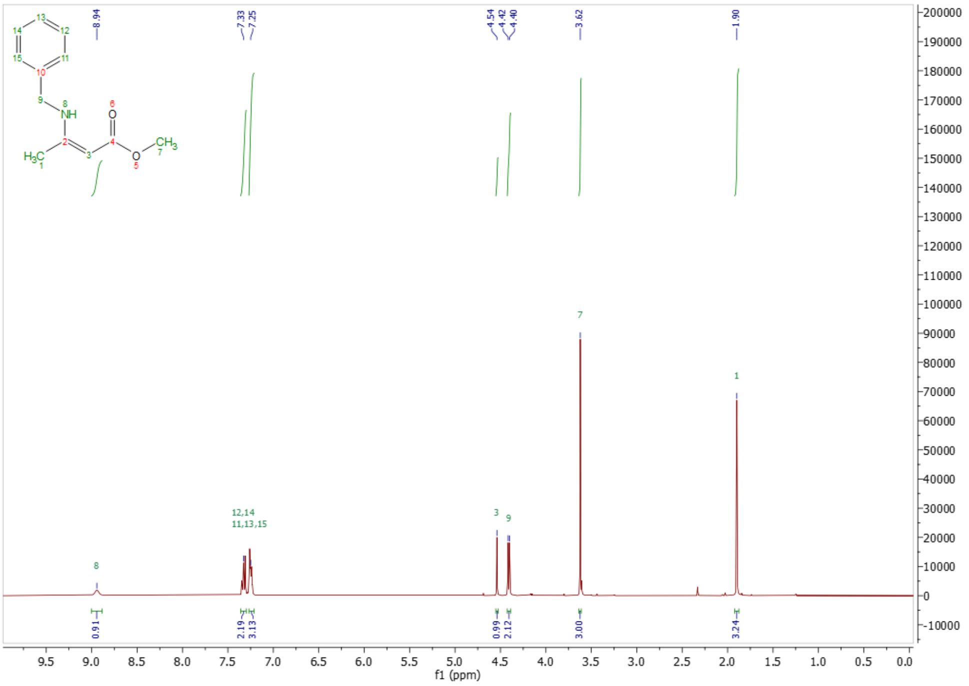

Fig. S14. ^{1}H NMR spectra of methyl 3-(benzylamino)but-2-enoate, **4**.

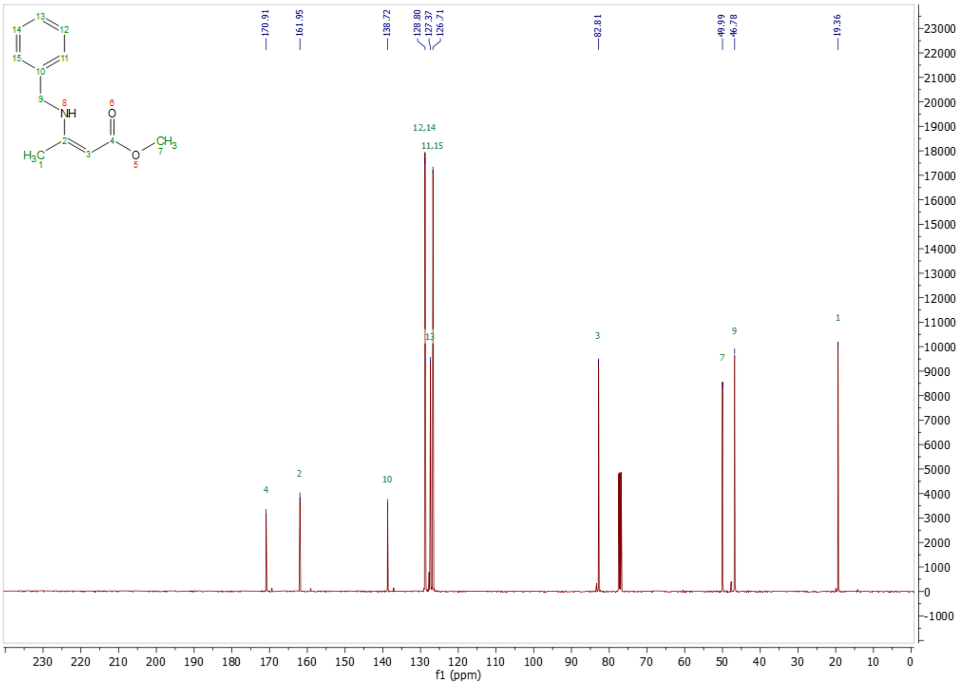

Fig. S15. ^{13}C NMR spectra of methyl 3-(benzylamino)but-2-enoate, **4**.

References

1 Acetonitrile - SAFETY DATA SHEET, https://www.sigmaaldrich.com/GB/en/sds/sial/271004?userType=undefined, (accessed 20 May 2025).

2 A. D. Clayton, A. M. Schweidtmann, G. Clemens, J. A. Manson, C. J. Taylor, C. G. Niño, T. W. Chamberlain, N. Kapur, A. J. Blacker, A. A. Lapkin and R. A. Bourne, *Chemical Engineering Journal*, 2020, **384**, 123340.

3 N. Aldulaijan, J. A. Marsden, J. A. Manson and A. D. Clayton, *React Chem Eng*, 2024, **9**, 308.

4 Y. Zhang, S. Tao, W. Chen and D. W. Apley, *Technometrics*, 2020, **62**, 291–302.

5 A. K. Kabi, R. Gujjarappa, N. Vodnala, D. Kaldhi, U. Tyagi, K. Mukherjee and C. C. Malakar, *Tetrahedron Lett*, 2020, **61**, 152535.

6 J. Vaitla, A. Bayer and K. H. Hopmann, *Angewandte Chemie - International Edition*, 2017, **56**, 4277–4281.
